# Early detection of drug-resistant *Streptococcus pneumoniae* and *Haemophilus influenzae* by quantitative flow cytometry

**DOI:** 10.1038/s41598-021-82186-4

**Published:** 2021-02-03

**Authors:** Takahiro Sawada, Masayuki Katayama, Shogo Takatani, Yoshiyuki Ohiro

**Affiliations:** 1 Fundamental Research Laboratory, Research and Development Division, Eiken Chemical Co., Ltd., 143 Nogi, Nogimachi, Shimotsuga-gun, Tochigi, 329-0114 Japan; 2 FCM Development, Business Strategy Development, Sysmex Corporation, 4-4-4 Takatsukadai, Nishi-ku, Kobe, 651-2271 Japan

**Keywords:** Diagnostics, Antimicrobial resistance, Clinical microbiology, Bacterial infection

## Abstract

Early detection of drug resistance contributes to combating drug-resistant bacteria and improving patient outcomes. Microbial testing in the laboratory is essential for treating infectious diseases because it can provide critical information related to identifying pathogenic bacteria and their resistance profiles. Despite these clinical requirements, conventional phenotypic testing is time-consuming. Additionally, recent rapid drug resistance tests are not compatible with fastidious bacteria such as *Streptococcus* and *Haemophilus* species. In this study, we validated the feasibility of direct bacteria counting using highly sensitive quantitative flow cytometry. Furthermore, by combining flow cytometry and a nucleic acid intercalator, we constructed a highly sensitive method for counting viable fastidious bacteria. These are inherently difficult to measure due to interfering substances from nutrients contained in the medium. Based on the conventional broth microdilution method, our method acquired a few microliter samples in a time series from the same microplate well to exclude the growth curve inconsistency between the samples. Fluorescent staining and flow cytometry measurements were completed within 10 min. Therefore, this approach enabled us to determine antimicrobial resistance for these bacteria within a few hours. Highly sensitive quantitative flow cytometry presents a novel avenue for conducting rapid antimicrobial susceptibility tests.

## Introduction

Emerging technologies such as semiconductor lasers and high-sensitivity sensors are anticipated to play a role in the early characterization of microorganisms^1–4^. Matrix-assisted laser desorption/ionization-time of flight mass spectrometry (MALDI-TOF MS) is now recognized as an innovative tool for identifying bacteria in a laboratory setting. Flow cytometry (FCM) is also a revolutionary tool that can analyze a huge number of cells at a single cell level in a short period. Therefore, various studies have focused on applying FCM to a clinical setting^5–7^.

Recently, the number of patients with lower respiratory tract diseases is increasing due to the worldwide progression of air pollution and aging^8,9^. *Streptococcus pneumoniae* and *Haemophilus influenzae* are the major causative bacteria of lower respiratory tract diseases. These bacteria are becoming a problem not only due to healthcare-associated infections but also due to an increase in resistant bacteria in community-acquired infections^9–14^.　Moreover, these bacteria can cause pneumonia, as well as serious diseases such as meningitis and sepsis^15^. To select an appropriate therapeutic drug, antimicrobial susceptibility tests (ASTs) must be carried out. These can reveal whether microbes are susceptible to antibiotics or resistant due to genetic mutations in penicillin-binding proteins or other factors^16–18^. Fast and appropriate antibiotic treatment of infectious diseases is important, especially in the case of sepsis. However, conventional phenotypic tests take 16–24 h from inoculation of the sample-processed and isolated-cultured bacteria to the return of result^19^. To obtain quick results, culture-free tests such as the polymerase chain reaction (PCR) have recently been applied; however, these methods can potentially miss resistance due to uncharacterized genes^10,20–23^. In addition to the treatment of diseases, a proper rapid resistance diagnosis may suppress the spread of antimicrobial-resistant bacteria^24,25^. Despite existing demand, there are few reports on phenotypic rapid ASTs for *S. pneumoniae* or *H. influenzae*^26,27^.

According to previous studies, it is critical to avoid interference from the components of the culture medium to establish a practical phenotypic AST^28,29^. Since *S. pneumoniae* and *H. influenzae* are fastidious, the Clinical and Laboratory Standards Institute (CLSI) has developed and standardized a test method for these species^30^. This method specifies that blood cell components or yeast extract should be added to the medium. However, debris such as dead bacteria and aggregates derived from these components reportedly interfere with specific and high-sensitivity FCM^31,32^. Therefore, in this study, we confirmed the feasibility of conducting specific and highly sensitive FCM analysis with bacterial strains that require a growth medium containing more nutrients than usual^33^.

In conventional studies using FCM, a method for predicting antimicrobial susceptibility has been investigated by comparing the ratio between plot groups consisting of scattered light or fluorescence intensity involved in cell activity^34,35^. Intercalators that permeate cell membranes, such as SYTO9, are used to measure whole bacterial counts. The impermeable intercalator propidium iodide which is taken up only by damaged bacteria is used to distinguish dead bacteria^32,33,35^. With the advent of quantitative FCM, the feasibility of applying FCM to drug susceptibility testing has recently been demonstrated^36,37^. However, these studies focused on bacteria that can grow in general media, such as *Escherichia coli* and *Pseudomonas aeruginosa*. As far as we know, detailed examinations of fastidious bacterial species have not yet been conducted^38^. Moreover, since the minimum inhibitory concentration (MIC) is considered according to a logarithmic distribution, consistent growth curves may not be obtained even if the same bacterial solution is used^39,40^.

We, therefore, combined quantitative FCM and a fluorescent intercalator to measure the time-dependent change in viable cells, using a few microliter samples from only 0.1 mL medium of the conventional microdilution method. Comparing the performance between the two, the feasibility of direct bacteria counting as a rapid phenotypical AST was validated.

## Results

### Viable cell counting from mixed medium solution

**Figure 1:**
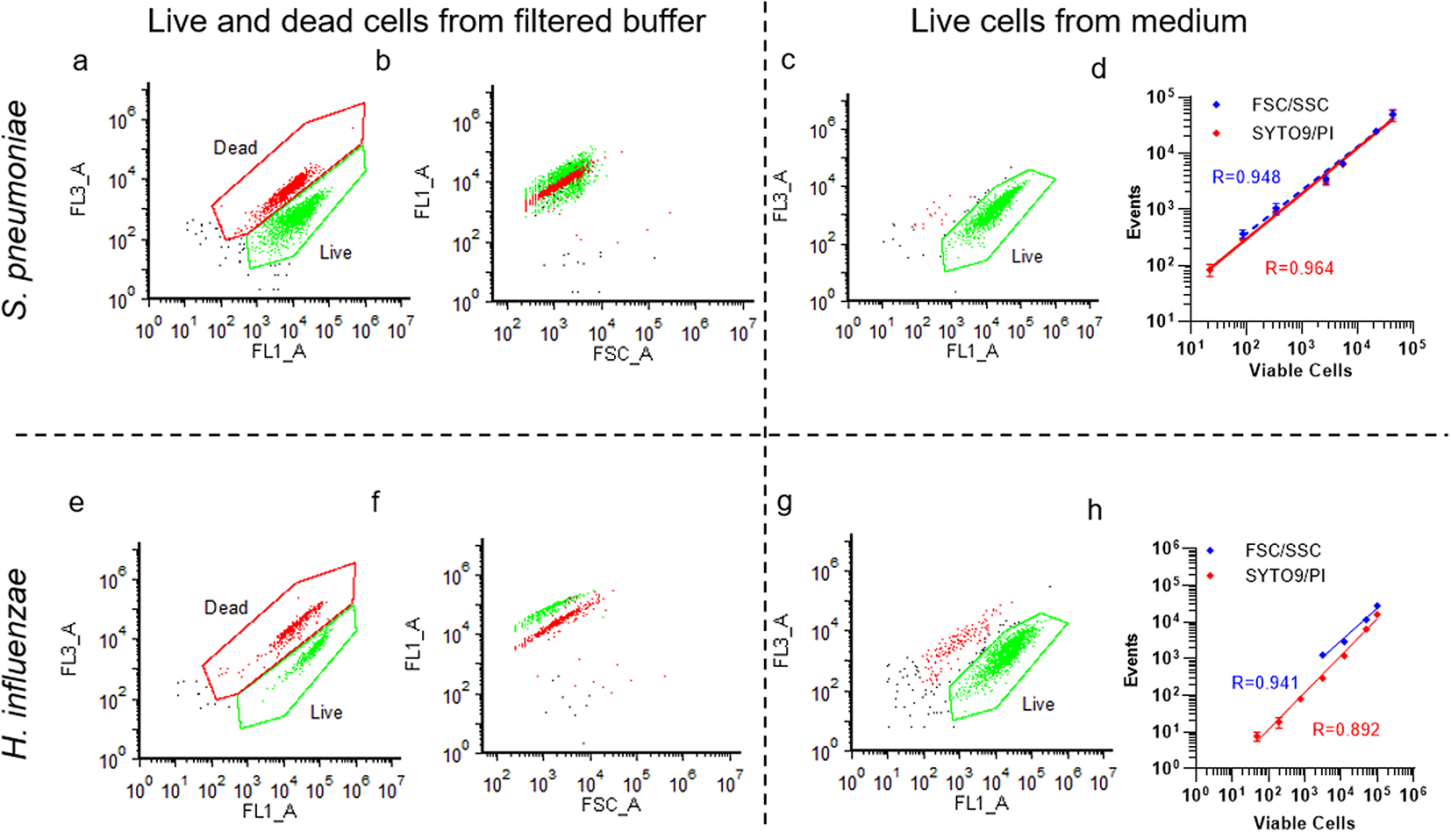
Correlation between flow cytometry (FCM) measurement and CFU measurement for *Streptococcus pneumoniae* and *Haemophilus influenzae*. Live (green) and dead (red) bacterial mixtures in the filtered buffer were SYTO9/PI stained and these plots were manually gated in FL1 and FL3 (**a**,**e**). The gated plots in the FL1-FL3 graph are colored and shown in the FSC-FL1 graph (**b**,**f**). Live (green) bacterial plots from medium contained lysed horse blood (**c**,**g**). (**d**, **h**) The number of viable cells was calculated based on the viable cell count obtained with CFU measurement. Events indicate the numbers of plots observed in the gate of 20 μL of the sample, as measured with FCM [number of experiments (N) = 3, error bars = 95% confidential interval]. The correlation is indicated in the logarithm. FSC/SSC blue circles are the results of measuring non-fluorescent-stained samples. SYTO9/PI red squares are the results of the fluorescent-stained. The plots that were not significantly different from the negative controls were not used for regression analysis and all plots are shown in Supplemental Fig. 1. The Spearman correlation coefficient (r-values) was obtained by regression analysis and is shown in each graph. Regression analysis was performed with weighted log–log nonlinear analysis. The obtained approximation formulae are shown as straight lines.

First, to validate the direct counting of bacteria in the medium by FCM, we analyzed the correlation between the numbers of bacteria and colony-forming units (CFUs). The target bacteria were *S. pneumoniae*, grown in a medium containing lysed horse blood, and *H. influenzae*, grown in a medium containing yeast extract and nicotinamide adenine dinucleotide*.* For live cell gating, we referred to a plot of a mixture of live and heat shock killed bacteria in a filtered buffer (Fig. 1a,e). The killed bacteria stained by propidium iodide (PI) had a stronger fluorescence intensity of FL3. In the case of *S. pneumoniae*, the fluorescence intensity by SYTO9 did not change with PI (Fig. 1b) unlike in *H. influenzae* (Fig. 1f). In the non-staining method, the plots of bacterial cells obtained using forward-scattered light and side-scattered light were single-gated. Therefore, the bacteria were counted as a whole number.

To determine the detection sensitivity, serial dilutions with a culture medium specific to the target bacterial species were prepared for both colony and FCM measurements. FCM was performed using a scattered light plot for non-staining bacteria and a fluorescence plot for staining bacteria. In the SYTO9/PI staining method, the gate indicating viable bacterial cells was obtained from the FL1 and FL3 plots (Fig. 1c,g).

Detection sensitivity was defined as the point at which a significant difference was obtained. This was determined using Dunnett's multiple comparison test with reference to the negative control. The concentration of *S. pneumoniae* (22 events/test; *P* = 0.0003) was detected with 4-fold more sensitivity using fluorescent staining than with the non-staining method (86 events/test; *P* < 0.0001). Since the FCM measurement used a diluted sample, the actual measured concentration was multiplied by the dilution rate. Therefore, using the fluorescence method, the sensitivity of counting the viable *S. pneumoniae* cells from the culture medium was found to be approximately 3 × 10^4^ CFU/mL*.* However, a substantial difference was not observed between the non-staining method for detecting all bacteria and the fluorescence method targeting only viable bacteria. In *H. influenzae*, the detectable point was 3099 events/test (*P* = 0.0004) for the non-staining method. The detectable point was identified with less sensitivity than that of *S. pneumoniae* due to the interference of contaminants in the medium at the gate of the scattered light plot. The fluorescent staining method maintained a sensitivity of 12 events/test (*P* = 0.0022), which was 2 × 10^4^ CFU/mL in terms of concentration in the culture medium. This sensitivity was equal to or higher than that observed for *S. pneumonia*. This suggests that the fluorescent staining method is 64-fold more sensitive than the non-staining method. The measured values of *H. influenzae* from the non-stained samples were affected by contaminants in the medium from yeast extract and nicotinamide adenine dinucleotide components (Supplemental Fig. 1). However, accurate whole bacterial cell counting was possible up to the maximum detectable points of the equipment (approximately 5 × 10^5^ events/test) using the non-staining method. Since the initial bacterial concentration via the general AST method is 5 × 10^5^ CFU/mL, these sensitivities of the fluorescent staining method were sufficient to reveal the changes in bacterial counts upon exposure to antimicrobial agents.

Nonlinear logarithmic regression analysis showed a high positive correlation between the CFUs measurement and FCM measurement, regardless of medium, species, and staining (Fig. 1d,h). Fluorescent staining and FCM measurement were completed within 10 min.

### Consistent antimicrobial susceptibility testing

Next, we sought to confirm the consistency of FCM and MIC with broth microdilution (BMD). We observed bacterial growth using FCM by cultivating bacteria in a medium containing an antimicrobial agent at certain concentrations. This allowed us to determine antibacterial resistance, as defined by the CLSI^19^. As shown in Table 1, *S. pneumoniae* ATCC 6303 was confirmed to be penicillin G-sensitive, while *S. pneumoniae* ATCC 49619 was penicillin G-resistant but cefotaxime-sensitive (PRSP: Penicillin G Resistant *S. pneumoniae*), and *S. pneumoniae* ATCC 700677 was resistant to both penicillin G and cefotaxime (MRSP: Multi-drug resistant *S. pneumoniae*). *H. influenzae* ATCC 49766 was susceptible to ampicillin and did not produce beta-lactamase. *H. influenzae* ATCC 33533 was resistant to ampicillin due to its beta-lactamase production but was susceptible to ampicillin-sulbactam. *H. influenzae* ATCC 49247 did not produce beta-lactamase but was resistant to both ampicillin and ampicillin-sulbactam.

**Table 1:** Antimicrobial resistance of the bacterial strains used in this study.

Species	Reference No	Antimicrobial	MIC (μg/mL)	Resistance	Beta-Lactamase	Detection time
*S. pneumoniae*	ATCC 6303	Penicillin G	0.0156	Susceptible	–	Not detected
*S. pneumoniae*	ATCC 6303	Cefotaxime	0.0156	Susceptible	–	Not detected
*S. pneumoniae*	ATCC 49619	Penicillin G	0.25	Resistance	–	180 min
*S. pneumoniae*	ATCC 49619	Cefotaxime	0.25	Susceptible	–	Not detected
*S. pneumoniae*	ATCC 700677	Penicillin G	8	Resistance	–	60 min
*S. pneumoniae*	ATCC 700677	Cefotaxime	2	Resistance	–	90 min
*H. influenzae*	ATCC 49766	Ampicillin	≤ 0.0625	Susceptible	Not detected	Not detected
*H. influenzae*	ATCC 49766	Sulbactam-Ampicillin	0.0156/0.0313	Susceptible	Not detected	Not detected
*H. influenzae*	ATCC 33533	Ampicillin	≥ 256	Resistance	Producing	120 min
*H. influenzae*	ATCC 33533	Sulbactam-Ampicillin	0.125/0.25	Susceptible	Producing	Not detected
*H. influenzae*	ATCC 49247	Ampicillin	4	Resistance	Not detect	120 min
*H. influenzae*	ATCC 49247	Sulbactam-Ampicillin	2/4	Resistance	Not detect	150 min

Antimicrobial resistance was determined from the minimum inhibitory concentration (MIC) based on the triple measurement obtained via broth microdilution (BMD). Penicillin G was the parenteral breakpoint, and ampicillin was the non-meningococcal breakpoint. The beta-lactamase was confirmed to be TEM-1 or ROB-1 by polymerase chain reaction (PCR) of *Haemophilus influenzae*. The detection time for *Streptococcus pneumoniae* was defined as the time when 60% or more bacterial growth was observed compared to the initial bacterial count. The detection time of *H. influenzae* was determined after at least 2 h of incubation, at which time the observed bacterial growth was compared to the bacterial count at 1 h of incubation.

Before FCM measurement, the bacterial suspensions were prepared and inoculated at approximately 5 × 10^4^ CFU/well on a microplate. This was done according to the standard BMD method. Subsequently, 5 $\upmu$L samples collected at different time points were stained in wells and measured by FCM, as described above. Initially, 500–1000 cells were measured for *S. pneumoniae* (equal to 6.5 × 10^5^ to 1.3 × 10^6^ CFU/mL) and 100–300 cells/events (1.3 × 10^5^ to 3.9 × 10^5^ CFU/mL) for *H. influenzae*.

**Figure 2:**
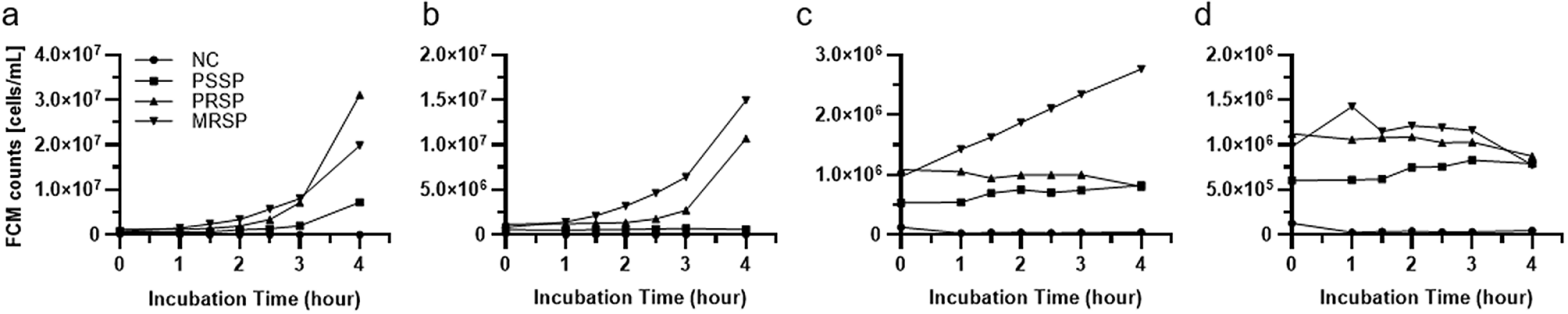
Growth curve of *Streptococcus pneumoniae* (**a**) and growth curve in 0.125 μg/mL penicillin G (**b**), 1 μg/mL cefotaxime (**c**), 2 μg/mL cefotaxime (**d**). All measurements were performed using samples stained with SYTO9 and propidium iodide PI. NC, negative control; PSSP, penicillin susceptible *S. pneumoniae*; PRSP, penicillin resistant *S. pneumoniae*; MRSP, multi-drug resistant *S. pneumoniae*. FCM counts were converted to CFU/mL by multiplying the number of events measured with FCM by the dilution rate.

Both PRSP and MRSP were grown in the wells containing penicillin G at breakpoint concentrations (Fig. 2).

Growth of the bacterial count was confirmed 1 h after inoculation, and resistance was confirmed. In cefotaxime, the results of FCM were also consistent with those of BMD. MRSP proliferation was only observed in 1 μg/mL cefotaxime; moreover, resistance was confirmed within 90 min. MRSP showed resistance to both penicillin G and cefotaxime. These results indicate that FCM could determine a non-susceptible bacterium in 90 min (Table 1).

**Figure 3:**
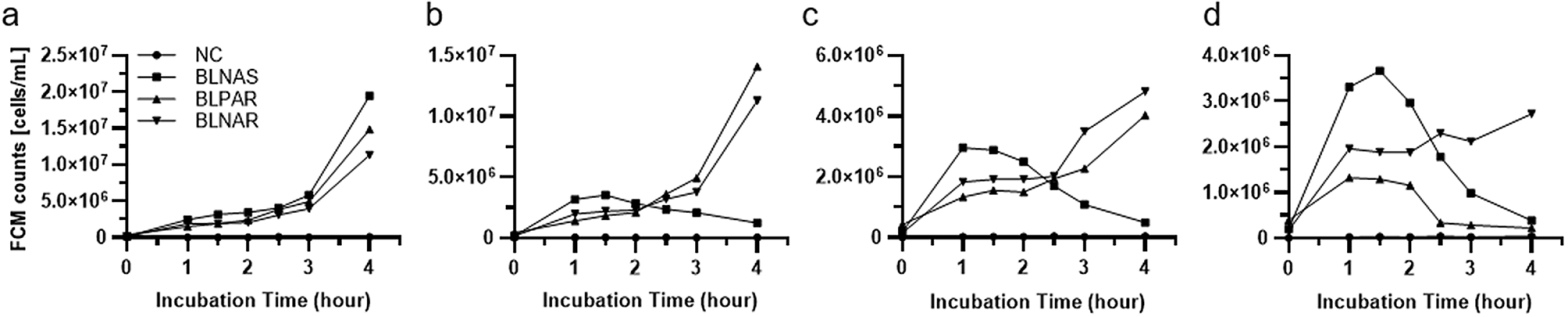
Growth curve of *Haemophilus. influenzae* (**a**) and growth curve in 1 μg/mL ampicillin (**b**), 2 μg/mL ampicillin (**c**), combination of 2 μg/mL ampicillin and 1 μg/mL sulbactam (**d**). All measurements were performed using samples stained with SYTO9 and propidium iodide PI. NC: negative control, BLNAS: beta-lactamase non-producing ampicillin-susceptible *H. influenzae*, BLPAR: beta-lactamase producing ampicillin resistant *H. influenzae*, BLNAR: beta-lactamase non-producing ampicillin resistant *H. influenzae*. FCM counts were converted to CFU/mL by multiplying the number of events measured with FCM by the dilution rate.

Regardless of the existence of an antimicrobial, all *H. influenzae* strains grew for 1–1.5 h after inoculation and then stagnated for 0.5–1 h (Fig. 3). Subsequently, after 2 h or more, they either died or proliferated. Therefore, the resistance was determined when the bacteria have grown compared to the number of bacteria 1 h after inoculation. Ampicillin resistance was detectable in both strains at 2 h, and resistance to the combination of ampicillin-sulbactam was detectable at 2.5 h (Table 1).

Incidentally, to confirm the effect of volume reduction due to multiple sampling from the same well, the remaining sample was finally cultured for up to 22 h. The resistance predicted from FCM measurements were consistent with the results of BMD.

## Discussion

In this study, we showed the feasibility of rapid and accurate counting of viable bacterial cells using highly sensitive and quantitative FCM. This method was coupled with a culture-based testing method and a nucleotide intercalator. Furthermore, we confirmed that this method can contribute to the rapid detection of antimicrobial resistance in bacteria. Surprisingly, clinically important resistance of *S. pneumoniae* could be detected within 1 h. This demonstrates that the culture time required to obtain results can be reduced by 95% or more compared with the conventional BMD method. Additionally, this method was able to detect the antimicrobial resistance of *S. pneumoniae* and *H. influenzae* earlier than the previously reported method of rapid measurement by FCM, which could only be applied to bacteria such as *E. coli*^36^.

Although there have been many reports of viable cell counting or monitoring using FCM since the 1980s, they were either clinically impractical due to the use of FCM equipment that lacked sensitivity or were non-quantitative^36,41^. These methods required complicated analytical logic and time-consuming sample preparation. Furthermore, there are no reports on viable cell counts of *Streptococcus* or *Haemophilus* species by FCM, especially the detection of antimicrobial resistance. By excluding the interference of contaminants from the culture medium, the method described here was able to count viable *S. pneumoniae* and *H. influenzae* cells with high sensitivity. Although the regression line between CFUs measurement and FCM counting shows high correlation values, FCM counting of *S. pneumoniae* have a tendency to be higher than CFU measurements. This may be because FCM counting were lower due to the effects of self-lysis during the test or frictional heat of smearing on the agar medium^42^. *H. influenzae* showed negative sections in fluorescent staining, suggesting that they were not completely stained because of media contaminants or adsorption on tubes while preparing dilution samples for FCM measurements^43^. Previous studies have revealed that^36,41^ this method enables the counting of both viable and dead cells of various bacterial species, such as *E. coli*, *P. aeruginosa*, *Staphylococcus aureus*, and *Enterococcus faecalis* in addition to species reported in this paper (data not shown). However, in the preliminary experiment as some plots of the dead cells prepared via heat shock disappeared, a precise measurement of the dead cells could not be conducted. The discrepancy in SYTO9 staining with PI between *S. pneumoniae*, a gram-positive coccus and *H. influenzae*, a gram-negative bacillus (Fig. 1b,f), would be related to factors such as membrane structures as well as membrane permeability^44^.

In this report, the resistance of *S. pneumoniae* and *H. influenzae* could be detected from 5 μL samples with a sensitivity of approximately 3 × 10^4^ CFU/mL or more. Since the initial bacterial concentration at the time of inoculation in the conventional BMD method is 5 × 10^5^ CFU/mL, our method requires a sample volume of 1 $\upmu$L or less per well using a 96-well microplate. As the influence of sampling is likely to be small, we can carry out early detection of antimicrobial resistance and confirm the results of culturing along with the conventional BMD method. In the future, detection accuracy can be further improved by using other fluorescent reagents, such as fluorescein isothiocyanate isomer, which can provide further information of proteins or enzymes in bacteria compared to intercalators only targeting for nucleic acids^45^.

As the performance of FCM equipment has recently improved, there have been studies measuring time-dependent bacterial changes or the accurate quantitative measurement of viable cells. However, most of these studies took single measurements per vial. On the other hand, our method allowed continuous measurement from one well and analysis of the details of proliferation events. For *S. pneumoniae*, bacterial growth could be detected within 1 h. It may be possible to detect growth even earlier than 1 h by increasing the number of measurement time points. Interestingly, we found that *H. influenzae* grew for approximately an hour after inoculation, regardless of being resistant or susceptible. This may be because of the post-antibiotic effect. Once bacteria were exposed to an antimicrobial agent, growth was inhibited even after removing the antimicrobial agent^46,47^. These phenomena suggest that monitoring the bacterial count is important for the reliable prediction of antimicrobial resistance^36^.

Although we confirmed the effects of volume reduction due to multiple sampling from the same microplate well using *S. pneumoniae* and *H. influenzae*, further studies need to be done with other strains or resistant strains. Moreover, since this method is based on BMD, it is necessary to pay attention to the MIC distribution, especially in the sub-MIC well. For example, the results of growth changes depend on the number of bacteria, compatibility with the medium, the number of plasmids in the bacteria, and other factors. It will, therefore, be necessary to combine molecular biological techniques in the future, such as the detection of resistance factors^48^.

In this study, we found that FCM presents a novel avenue for conducting rapid ASTs. This platform may be advanced with technological innovations such as FCM imaging. With these technologies, further improvements in infectious disease treatment and suppression of drug-resistant bacteria are expected.

## Methods

### Strains and target antimicrobials

Three strains each of *S. pneumoniae* and *H. influenzae* were used: one antimicrobial-susceptible strain and two resistant strains were prepared. Viable cell counting was performed using *S. pneumoniae* ATCC 49619 and *H. influenzae* ATCC 49766, which are used as control strains in CLSI testing^19^.

To validate the early detection of resistance by FCM, the following antimicrobials were selected: 0.125 μg/mL penicillin G and 1–2 μg/mL cefotaxime (a cephalosporin); moreover, they were tested against *S. pneumoniae* ATCC 49619, ATCC 6303, and ATCC 700677^19,49,50^. *H. influenzae* ATCC 49766, ATCC 33533, and ATCC 49247 were tested with 1–2 μg/mL ampicillin and a combination of 2 μg/mL ampicillin and 1 μg/mL sulbactam, which served as an ampicillin and beta-lactamase inhibitor^51,52^. *S. pneumoniae* ATCC 49619 and ATCC 6303 were purchased from Kanto Kagaku (Tokyo, Japan). Other strains were purchased from the American Type Culture Collection (ATCC; Manassas, VA, USA).

### Confirmation of antimicrobial resistance

The antimicrobial resistance and MIC profiles of the strains were determined by the manual method, according to the BMD described in the CLSI criteria^30^. Cation-adjusted Mueller–Hinton broth (Becton Dickinson, MD, USA), which had lysed horse blood added to a final concentration of 5%, was used as a medium for *S. pneumoniae*. The *Haemophilus* test medium for *H. influenzae* was used as described in the CLSI criteria. This medium was prepared using cation-adjusted Mueller–Hinton broth, nicotinamide adenine dinucleotide*,* and yeast extract*.* The production of *H. influenzae* beta-lactamase was confirmed by PCR using previously reported primers synthesized by Eurofins Genomics (Tokyo, Japan)^53^.

### Counting of colony forming units

For CFUs measurement, samples of 100 to 300 CFU/20 μL were selected from the dilution series used for FCM quantitative viable cell counting, described later, and directly applied to different agar media for each bacterial species. *S. pneumoniae* strains were cultured in 5% sheep blood agar medium (Eiken Chemical, Tochigi, Japan) for 18 h at 35 °C. *H. influenzae* was cultured in chocolate agar medium (Eiken Chemical, Tochigi, Japan) in 5% CO_2_ at 35 °C. The number of cultured colonies was multiplied by the dilution ratio and used as a standard for determining the number of viable bacteria.

### Quantitative viable cell counting

*S. pneumoniae* strains were precultured in 5% sheep blood agar medium (Eiken Chemical, Tochigi, Japan) for 18 h at 35 °C. *H. influenzae* was precultured in chocolate agar medium (Eiken Chemical, Tochigi, Japan) in 5% CO_2_ at 35 °C. The obtained colonies were suspended in sterile saline (0.35% NaCl) and adjusted to MacFarland 1.0 by measuring absorbance (620 nm wavelength). The bacterial suspensions were serially diluted fourfold with phosphate-buffered saline (PBS; 20 mM phosphate buffer, 130 mM NaCl, pH 7.4) using a glass tube. Diluted bacterial suspensions (5 μL) were further diluted with PBS in a 96-well Nunc-Immuno Module plate microplate (Thermo Fisher Scientific, Waltham, MA, USA). Using this microplate, FCM measurement was performed. For fluorescent staining, the samples were dispensed in a microplate containing PBS with 5 μM SYTO9 and 15 μM PI. Samples were subsequently incubated at room temperature in the dark for 5 min. FCM measurement was performed after fivefold dilution with PBS to suppress background fluorescence. All fluorescent staining reagents were purchased from Thermo Fisher Scientific (Waltham, MA, USA). FCM measurement was performed using RF-500 (Sysmex, Kobe, Japan) equipped with a blue semiconductor laser (488 nm wavelength). The built-in front scattering (FSC) and side scattering (SSC) detectors were used to detect scattered light. An FL1 filter (527+/−15 nm, SYTO9) and FL3 filter (695+/−25 nm, PI) was used for fluorescence detection. The measurement volume was set to 20 μL and the flow rate was 1.85 μL/s.

### Flowcytometric viable cell counting

For viable cell monitoring by FCM, a 5 μL sample was collected from a 100 μL medium containing the bacteria. The sample was taken from the same microplate well of BMD and mixed with PBS containing SYTO9 (5 μM) and PI (15 μM). Next, each sample was incubated at room temperature in the dark for 5 min. FCM measurement was performed after 5-fold dilution with PBS to suppress background fluorescence. The microplate with inoculated wells was immediately returned to the incubator to restart the culture.

The FCM measurement was operated on RF-500 software (Sysmex, Kobe, Japan). Bacterial counting was performed using FCS Express 6 RUO Edition (De Novo Software, Los Angeles, CA, USA).

### Statistical analysis

The detection limits of the viable cell counting were analyzed by Dunnett's multiple comparison test. This test was used to compare the measured value at each time point and the negative control value using StatFlex Ver7.0 (Artec Inc., Osaka, Japan). In these analyses, a *p* value less than 0.05 was considered significant. Regression analysis of the viable cell counts was performed with the GraphPad Prism 6 software (GraphPad Software Inc., San Diego, CA, USA).

## Supplementary Information

Supplementary Figure 1.
