# Supplementary material for: Early detection of drug-resistant Streptococcus pneumoniae and Haemophilus influenzae by quantitative flow cytometry
Source: Sci Rep. 2021 Feb 3;11:2873. doi: 10.1038/s41598-021-82186-4 (PMC7859230; doi:10.1038/s41598-021-82186-4)
Supplement: Supplementary file 1 — Supplementary Figure 1. [file 41598_2021_82186_MOESM1_ESM.pdf]

Early detection of drug-resistant *Streptococcus pneumoniae* and *Haemophilus influenzae* by quantitative flow cytometry

Takahiro Sawada[1], Masayuki Katayama[2], Shogo Takatani[2], Yoshiyuki Ohiro[1*]

Supplemental Figures

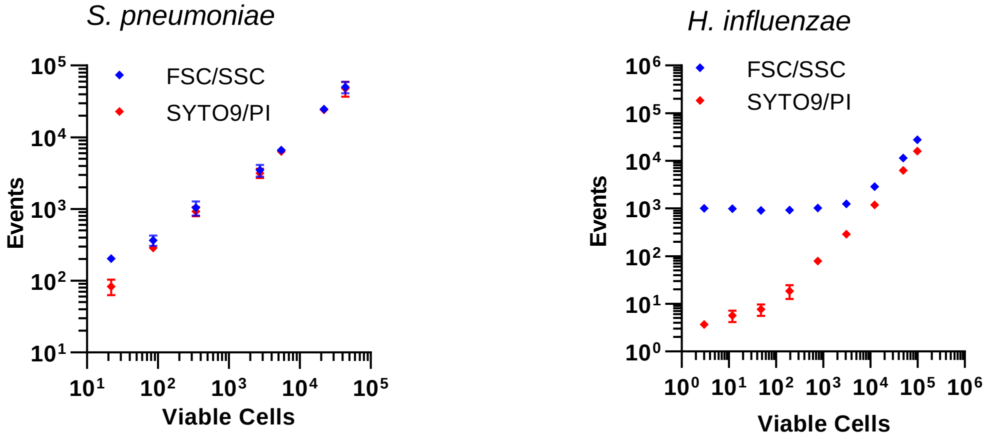

Supplemental Figure 1. Correlation between flow cytometry (FCM) analysis and culture methods for *Streptococcus pneumoniae* and *Haemophilus influenzae*.

Viable cells were calculated based on the viable cell count obtained by the CFU measurement. Events indicate the numbers of plots observed in gate of 20 μL of the sample, as measured by FCM [number of experiments (N)=3, error bars = 95% confidential interval]. The correlation is indicated in the logarithm. FSC/SSC ● are the results of measuring non-fluorescent-stained samples. SYTO9/PI ■ are the results of fluorescent sample stained.
